# Stress and resilience: cortisol hypo-response to acute stress in non-resilient individuals

**DOI:** 10.1007/s00702-026-03218-9

**Published:** 2026-07-03

**Authors:** Nadine D. Riske, Charlotte Schenk, Kira F. Ahrens, Bianca Kollmann, Danuta Weichert, Nadine Steinbach, Jana F. Burlage, Florian Freudenberg, Klaus Lieb, Raffael Kalisch, Oliver Tüscher, Maren Schmidt-Kassow, Andreas Reif, Michael M. Plichta

**Affiliations:** 1 Department of Psychiatry, Psychosomatic Medicine and Psychotherapy, University Hospital, Goethe University Frankfurt, Frankfurt, Germany; 2 Cooperative Brain Imaging Center - CoBIC, Goethe University Frankfurt, Frankfurt, Germany; 3 Department of Psychiatry and Psychotherapy, University Medical Center of the Johannes Gutenberg University, Mainz, Germany; 4 Leibniz Institute for Resilience Research, Mainz, Germany; 5 Institute of Molecular Biology, Mainz, Germany; 6 Neuroimaging Center, Focus Program Translational Neuroscience, Johannes Gutenberg University Medical Center, Mainz, Germany; 7 Department of Neuropsychology and Psychological Resilience Research, Central Institute of Mental Health, Mannheim, Germany; 8 Department of Psychiatry, Psychotherapy and Psychosomatic Medicine, University Medicine Halle (Saale) of the Martin Luther University Halle-Wittenberg (MLU), Halle (Saale), Germany; 9 German Center for Mental Health (DZPG), Partner Site Halle-Jena-Magdeburg, Halle (Saale), Jena, Magdeburg, Germany

**Keywords:** Resilience, Cortisol, Stress, Hypothalamic-pituitary-adrenal axis

## Abstract

Resilience—the outcome of effective adaptation to adversity—is a dynamic construct referring to the preservation or quick recovery of mental health in the face of significant stressors. Despite the crucial role of the acute stress response in coping with adversity, no consensus exists regarding its relationship with resilience. Inconsistent findings may be due to variability in the assessment of resilience. The present study investigates the relationship between resilience and acute stress responses by comparing resilient and non-resilient individuals in their responses to experimentally induced acute stress. A novel approach was used, combining the Socially Evaluated Cold Pressor Test (SECPT) as a reliable and replicable stress induction method with a uniquely characterized sample whose resilience had been assessed in a prior longitudinal study. Based on these longitudinal assessments of stressor reactivity, participants (*N* = 48) were classified as resilient (14 female / 10 male; age in years: *M* = 34.5, *SD* = 9.32) or non-resilient (17 female / 7 male; age in years: *M* = 31.6, *SD* = 5.52). Salivary cortisol and subjective measures (subjective stress and State Anger) were assessed before and 20 min after stress induction. Resilience was associated with cortisol reactivity, with non-resilient participants showing significantly lower cortisol responses to the SECPT compared to the resilient participants (*F*(1,45) = 4.252, *p*= 0.045). Subjective measures did not differ between groups in terms of reactivity. However, non-resilient participants reported higher overall levels of subjective stress (*F*(1,43) = 4.780, *p*= 0.034). It remains to be clarified whether resilience determines cortisol reactivity or whether cortisol reactivity represents a mechanism underlying resilience.

**Supplementary Information:**

The online version contains supplementary material available at https://doi.org/10.1007/s00702-026-03218-9.

## Introduction

According to the World Health Organization (WHO), stress can be seen as a natural, activating and adaptive reaction of body and mind to challenging or threatening situations (World Health Organization, 2024). Chronic or excessive stress can affect physical health, as it is associated with various diseases (Russell and Lightman 2019; Yang et al. 2015), with the stress hormone cortisol playing an important role (Knezevic et al. 2023). Cortisol is frequently used as biological marker for stress (Levine et al. 2007), which normally increases in response to stress (Russell and Lightman 2019). Its secretion is regulated by the hypothalamic-pituitary-adrenal (HPA) axis (Thau et al. 2024), which is considered the principal element of the physiological stress response (Fries et al. 2009). Apart from its physical impact, stress is also a relevant factor in the development of mental disorders (Kendler et al. 1999; Palamarchuk et al. 2023). Mental disorders are widespread worldwide and contribute significantly to disability-adjusted life-years (GBD 2019 Diseases and Injuries Collaborators 2020, 2022). Remarkably, nearly everyone encounters stressful circumstances eventually during life, but not everyone develops mental illness as a result (McEwen and Stellar 1993). The attempt to explain this variability in stress response leads to the construct of resilience.

Resilience can be understood as the outcome of effective adaptation to adversity and as a dynamic concept referring to the preservation or quick recovery of mental health during and after experiencing significant stressors (Bonanno et al. 2011; Kalisch et al. 2017).

HPA axis reactivity might be a relevant mechanism in the context of resilience, since it is known to be dysregulated under certain circumstances. Dysregulations of cortisol reactivity to experimental stress are caused by many factors, and in different directions (Foley and Kirschbaum 2010). Considerable alterations in HPA axis functioning in humans have been shown in relation to chronic stress, including both hyper- and hypoactivity reflecting complex patterns of dysregulation (Miller et al. 2007). Excessive chronic stress can change the circadian rhythm of the HPA axis and cause dysregulated cortisol secretion (McEwen and Gianaros 2010). As part of a complex interplay of molecular processes, stress-induced, prolonged cortisol release might lead to glucocorticoid resistance (Zefferino et al. 2021), and over time, to desensitization of the HPA axis and a blunted cortisol response (Knezevic et al. 2023). The association between early-life stress exposure and blunted cortisol reactivity (Young et al. 2021) may suggest that vulnerability is linked to a hypo-responsive cortisol profile. Further, a link seems to exist between post-traumatic stress disorder (PTSD) and alterations of the HPA axis, even if the exact relationship is still unclear. Even though some studies have found no difference in HPA reactivity compared to healthy controls (Majewski et al. 2023; Roelofs et al. 2009; Simeon et al. 2007), there is strong evidence for cortisol hypo-reactivity in PTSD patients in response to an acute stressor (Metz et al. 2020; Wichmann et al. 2017; Zaba et al. 2015). Overall, the predominant finding is a dysregulated HPA axis in PTSD patients, characterized by lower cortisol output and hypo-responsiveness (Morris et al. 2012). However, as our study examined healthy individuals with varying levels of resilience, direct generalization from PTSD populations remains unclear. Moreover, findings within the PTSD literature are not entirely consistent, with evidence for both hypo- and hyper-responsiveness, which led us to formulate non-directional hypotheses.

The acute stress response has been considered the principal driver of adaptation to adversity and is therefore of fundamental importance for understanding resilience (Hermans et al. 2025). The relationship between resilience and the acute stress response has not yet been sufficiently clarified. A connection has thus far only been found between resilience and acute subjective stress response, but not for physiological stress response as measured by salivary cortisol (García-León et al. 2019). However, it has been shown that higher resilience in young men was related to lower cortisol baseline levels and lower cortisol output overall, although highly and less resilient individuals did not differ significantly regarding their cortisol reactivity (Mikolajczak et al. 2008). In caregivers of autism patients, high resilience was associated with lower cortisol release (Ruiz-Robledillo et al. 2017). Greater anticipatory increases in cortisol in response to acute stress were associated with better mental health outcomes following adversity (Shao et al. 2023).

A study on police officers undergoing experimental stress followed by four years of observation during active-duty service revealed that cortisol reactivity predicted different trajectories regarding resilience (Galatzer-Levy et al. 2014). High cortisol reactivity was related to resilient trajectories while low cortisol reactivity indicated an increased risk of developing chronic non-remitting stress responses.

Concerning the relationship between physiological and subjective response to acute stress, there is no consensus. While salivary cortisol and subjective stress assessed directly after stress induction were not associated, recent high stress exposure increased the probability of higher acute cortisol responses (Dalile et al. 2022). Acute physiological and subjective stress response might be inverted, since high subjective stress scores appear to be associated with low general salivary cortisol levels after stress induction (Buzgoova et al. 2020).

Inconsistencies in the literature on the relationship between resilience and acute subjective and physiological stress responses may reflect the complexity and variability in the measurement of resilience, with mainly short-term (García-León et al. 2019; Mikolajczak et al. 2008; Rösner et al. 2023; Ruiz-Robledillo et al. 2017) and scarcely any long-term (Chmitorz et al. 2021) approaches existing. Resilience is not a permanent state or character trait, but rather a dynamic concept that changes over time and can be seen as the result of adaptation to challenging experiences (Kalisch et al. 2017, 2019; Rutter 2012). Questionnaires are not suitable for accurately predicting resilience (Bonanno 2021). Its investigation requires longitudinal research approaches (Kalisch et al. 2017).

According to the Longitudinal Resilience Assessment (LORA) study (Chmitorz et al. 2021), up to five clusters have been identified that represent distinct temporal profiles of stressor reactivity (SR) following a major life event (Ahrens et al. 2024). SR reflects how mental health changes in relation to stressor exposure, defined as the deviation from the expected association between stress exposure and mental health problems in the study sample (Kalisch et al. 2021). Positive SR values indicate more, and negative values fewer mental health problems than expected given the level of stressor exposure.

**Fig. 1:**
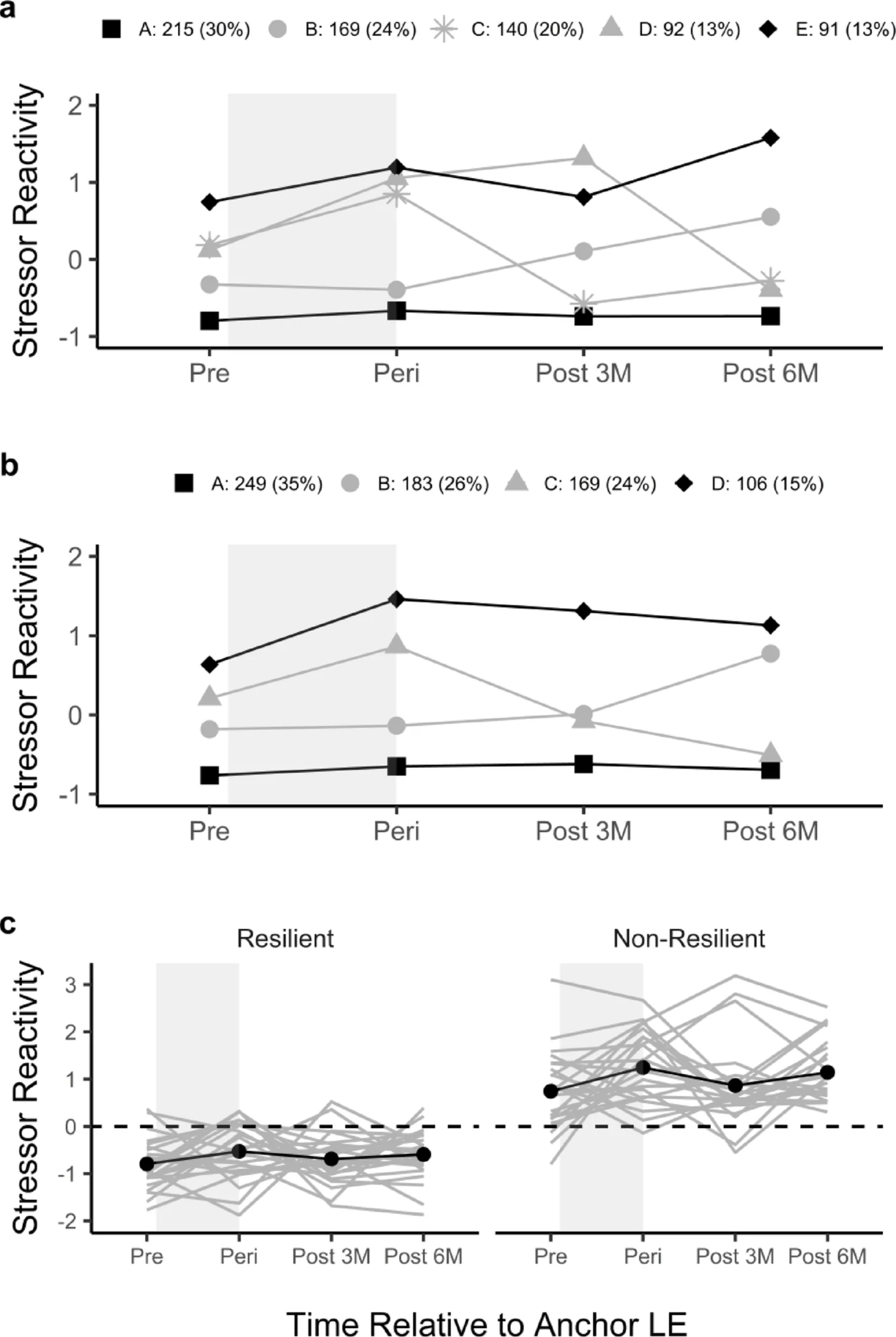
Distinction of resilient and non-resilient participants based on Stressor Reactivity (SR) before (Pre), during (Peri), 3 months after (Post 3 M), and 6 months after (Post 6 M) a life event (LE), showing **a** the 5-cluster solution, **b** the 4-cluster solution, and **c** individual SR trajectories of the present study sample. Numbers above indicate the number of participants per cluster in **a** and **b**. In the 5-cluster solution (**a**), clusters are characterized as follows: (A) consistently low SR, (B) delayed onset of increased SR, (C) increased SR followed by rapid recovery, (D) increased SR followed by delayed recovery, and (E) chronically elevated SR. **a** and **b** have been previously published (Ahrens et al. 2024), © 2024 The Author(s)

The five identified trajectories, as illustrated in Fig. 1a, can be functionally characterized as follows: Cluster A shows consistently low and stable SR over time. Individuals in this cluster are referred to as ‘resilient’ in the following. Cluster B is characterized by a delayed increase in SR following the life event, indicating a delayed stress response. Cluster C shows an initial increase in SR followed by rapid recovery, whereas Cluster D is characterized by an initial increase followed by delayed recovery. Cluster E shows persistently elevated SR over time, reflecting chronically increased stress-related mental health problems. We use the term ‘non-resilient’ to describe participants constantly presenting a high level of SR because these individuals deviate most strongly from the largest, ‘resilient’ cluster, and because in this cluster indications of mental illness were found significantly more often in Mini-International Neuropsychiatric Interviews (MINI; Lecrubier et al. 1997; German version: Ackenheil et al. 1999), a screening tool for mental disorders. Importantly, there is not only one “resilient” cluster, as resilience is represented across several clusters. For example, Cluster C may also be considered resilient, as it reflects a temporary increase in SR followed by rapid recovery to baseline levels.

The present study investigates the relationship between resilience and the acute physiological and subjective stress response by comparing resilient and non-resilient individuals with respect to their response to experimentally induced acute stress. Since resilience can be associated with both hypo- or hyper-responsiveness to acute stress, the hypotheses are formulated non-directional:

1. Resilient and non-resilient adults differ in their acute physiological stress response, represented by salivary cortisol.
2. Resilient and non-resilient adults differ in their acute subjective stress response, represented by subjective stress and State Anger.

## Experimental procedures

### Study design

The present study, which is hereafter referred to as StReak study (a portmanteau of ‘stress’ and ’reaction’), used an interventional mixed design, with the between-subject factor *group* (resilient vs. non-resilient) and within-subject factor *time* (pre- vs. post-stress induction). Prior to data collection, a research protocol was created. The protocol included the study design, hypotheses, sample size considerations, outlier identification procedures, and the planned analyses. It served as an internal project record and was not publicly registered. A PDF version of this pre-data-collection protocol has been retained and is available from the corresponding author upon reasonable request.

### Participants

All participants were recruited from the sample of the LORA study (Chmitorz et al. 2021). Thus, the StReak study is based on longitudinal observation of resilience over approximately 4.5 years. Formation of clusters was determined prior to the StReak study as it is based on findings of the LORA study (Ahrens et al. 2024; Chmitorz et al. 2021), including the development of diverse cluster solutions with varying numbers of clusters (Fig. 1a + b). The StReak study focused on the 5-cluster solution, which demonstrated the best model fit and theoretical meaningfulness (Ahrens et al. 2024). Recruitment involved participants classified as showing a ‘resilient’ trajectory (Fig. 1a, Cluster A – consistently low SR) and a ‘non-resilient’ trajectory (Fig. 1a, Cluster E – chronically elevated SR). To achieve the necessary statistical power, recruitment of non-resilient participants was extended to the corresponding cluster in the 4-cluster solution (Fig. 1b, Cluster D – highest SR in that solution).

Participants were initially planned to be recruited based on the 5-cluster solution. However, as Cluster E (chronically elevated SR) included only 91 participants, we additionally included participants from the corresponding cluster in the 4-cluster solution (including 106 participants) to ensure an adequate sample size. Most non-resilient participants (20 of 24) had been assigned to Cluster E in the 5-cluster solution. The remaining 4 had been assigned to Cluster D in both the 4-cluster and the 5-cluster solution. Resilient participants were recruited exclusively from Cluster A in both solutions. The individual time profiles on which the clusters are based were reproduced for our study sample (Fig. 1c) to demonstrate that the groups differ clearly, and that the stress reactivity trajectories resemble the original clusters.

The final sample of *N* = 48 participants consisted of two groups: resilient (14 female / 10 male; age in years: *M* = 34.5, *SD* = 9.32) and non-resilient (17 female / 7 male; age in years: *M* = 31.6, *SD* = 5.52). We aimed for an equal distribution in both groups regarding sex and handedness. To ensure the absence of current mental disorders, the MINI was considered before reaching out. Potential volunteers underwent a phone screening. Inclusion criteria were participation in LORA study, German level C1, and age between 18 and 55 years. The following led to exclusion from the study: pregnancy, any neurological or psychological disorder, any serious physical constraint (especially cardiological diseases), strong skin irritation on the hand area, fibromyalgia, heavy smoking (more than 10 cigarettes per day), high intake of coffee (more than 600 ml/day), alcohol consumption in the last 14 h, and diseases and systemic medication influencing the HPA axis, with the exception of five individuals takingL-tyroxine (see Supplementary Material for more details). The study was approved by the Ethics Committee of the Goethe University Frankfurt (2024 − 2012). Participants provided written informed consent regarding participation and publishing their data. Participation was voluntary and compensated financially with 20 euro per hour. The study was conducted in accordance with the principles of the Declaration of Helsinki of 2024 (World Medical Association, 2024).

### Stress induction

The experiment was conducted in the Psychophysiology Laboratory at the Department of Psychiatry, Psychosomatics and Psychotherapy, University Hospital Frankfurt in a single, approximately one-hour session. Acute stress was induced using the Socially Evaluated Cold Pressor Test (SECPT; Schwabe et al. 2008). All participants were tested under the same conditions, as the SECPT was performed identically for everyone using cold water (3–5 °C) and by the same female experimenter, who did not know any of the participants. During the SECPT, participants immersed the right hand up to the wrist in cold water for as long as possible—up to three minutes—while being continuously observed by the experimenter sitting opposite, with a neutral facial expression. Participants were informed that a camera was recording the task and were instructed to look into the camera without speaking. In the final debriefing, they were informed that no recording had taken place. Mean value of water temperature, recorded before and after stress induction, was documented. Measurements were obtained at two points to compare within-person changes between groups before (T0) and after (T1) stress induction.

### Physiological response: salivary cortisol

Cortisol concentrations were assessed in saliva samples. As pulsatile cortisol release is influenced by circadian rhythms, saliva samples should be collected at comparable times (Lee et al. 2015). Thus, saliva was collected between 2 and 6 pm (with the exception of two individuals whose saliva was unintentionally taken 6 and 11 min too early), consistent with similar research (Mikolajczak et al. 2008). Since their baseline cortisol level neither differed significantly from reference values (Miller et al. 2016) nor changed results of analyses, these values were included. Participants completed a preliminary control questionnaire to identify factors influencing cortisol levels such as sex, age, sleep, medication, and use of coffee, alcohol, and nicotine. Menstrual cycle was recorded in female participants by a questionnaire, given that cortisol level is higher in the luteal phase than in the follicular phase (Kirschbaum et al. 1999). To guarantee high quality and validity of saliva samples, participants were instructed to abstain from drinking water, eating, using chewing gum, or exercising for one hour prior to the appointment. Saliva was collected directly before (T0) and exactly 20 min after (T1) the start time of the SECPT (defined by hand immersion in cold water), consistent with findings on maximum cortisol level after stress induction (Gordis et al. 2006; Schwabe et al. 2008). Saliva was gathered by the participant placing a cotton swab into their own mouth for one minute and then stored refrigerated in a tube until analysis.

### Subjective response: subjective stress and State Anger

To assess subjective stress, defined as the participants’ momentary self-reported stress experience, a Visual Analogue Scale (VAS) was used at both time points. Participants were instructed to indicate their current level of subjective stress by placing a mark on a 10-cm horizontal line. The endpoints were labeled “not stressed” (accompanied by a neutral smiley face) and “very stressed” (accompanied by a smiley face). The VAS version is provided in the Supplementary Material. The VAS was chosen due to its simplicity, brevity, and suitability for repeated assessment of momentary subjective stress in experimental settings. It is widely used in acute stress research and has been shown to be sensitive to stress induction procedures (Hellhammer and Schubert 2012), with evidence supporting its construct validity and associations with established stress questionnaires (Lesage et al. 2012). The State-Trait Anger Expression Inventory-2 (STAXI-II) questionnaire (Spielberger 1999; German version: Rohrmann et al. 2013), a tool for measuring anger as an emotional state (State Anger) and personality trait (Trait Anger), is used to assess a person’s tendency towards aggression. In the StReak study, only the factor of State Anger was assessed using the first part of the STAXI-II, at both times. Due to technical issues, two values are missing for subjective stress and State Anger (*N* = 46).

### Data processing

Raw data was compiled and comparability of groups concerning age, sex, and handedness was evaluated. Saliva samples were pre-processed using centrifugation in the laboratory of the Department of Psychiatry, Psychosomatics and Psychotherapy at the University Hospital in Frankfurt. Cortisol levels (in nmol/L) were determined for every participant in duplicates using the Cortisol Saliva Luminescence Immunoassay according to the manufacturer’s instructions (IBL INTERNATIONAL GMBH 2022). VAS for subjective stress was evaluated with a ruler measuring the distance between left end of scale and the crosses marked by the participants. The STAXI–II questionnaire was analyzed according to the user manual (Rohrmann et al. 2013). Baseline cortisol concentrations were compared to reference ranges for salivary cortisol generated for males and females in different age and daytime categories (Miller et al. 2016).

### Statistical analysis

Statistical analyses were performed using IBM SPSS statistics (version 29). Box plots were generated to identify possible outliers for all variables. Both time points before (T0) and after (T1) stress induction, as well as their difference (T1-T0), were plotted for groups separately. All variables were tested for normal distribution using the Kolmogorov-Smirnov test. Repeated measures analyses of variance (ANOVAs) with between-subject factor group (resilient vs. non-resilient) and within-subject factor time (pre- vs. post-stress induction) were conducted separately for salivary cortisol, subjective stress, and State Anger. Since the within-subject factor time has only two levels, sphericity is given as a criterion for ANOVA. Repeated measures ANOVAs are robust to violations of the normality assumption (Blanca et al. 2017). *P* ≤ 0.05 was considered as significant. Partial eta squared (ηp2) is reported to indicate effect size. The duration of hand immersion in cold water (SECPT time) was included as a covariate in all analyses (Manigault et al. 2021), given its importance for interpreting group differences in stress reactivity. This is especially important because non-resilient individuals may exhibit reduced pain tolerance (Hemington et al. 2017), potentially affecting SECPT immersion time.

To evaluate group comparability, we recorded participants’ age, sex, and handedness, as well as lifestyle and physiological variables, including frequent coffee consumption (> 400 ml/day), alcohol use on the previous day, nicotine use (occasional or regular), and, for female participants, oral contraceptive use and menstrual cycle phase (follicular vs. luteal). All variables were statistically tested for differences between groups using Chi-square test or Mann–Whitney U test (see Table 1).

Due to non-normally distributed cortisol data (right-skewed), a log10 transformation was performed. Following this, the cortisol data conformed to a normal distribution. This was true for both time points and for both samples. To illustrate individual cortisol trajectories over time, spaghetti plots were created. Correlation analyses were conducted using R (version 4.3.3) to examine associations between difference scores (T1-T0) for all measures, across all groups and for both groups separately. Spearman’s rank-order correlations (ρ) were computed for all correlation analyses.

As part of additional exploratory analyses, the association between acute and long-term physiological stress responses was examined by correlating salivary cortisol with hair cortisol concentration (HCC). Analyses were conducted in a subsample of 26 participants (12 resilient / 14 non-resilient) with available HCC data across all participants and within groups.

## Results

Group comparisons presented in Table 1 revealed no significant differences. Mean, standard deviation (*SD*), and range for all measures, each at T0, T1, and concerning the difference (T1-T0) are presented in Table 2. Cortisol values at both time points were non-normally distributed (all Kolmogorov–Smirnov *p*< 0.20) and right-skewed. Therefore, a log10 transformation was applied, resulting in normally distributed data across the total sample and within both groups (all p values > 0.20). Based on outlier analyses (see Supplementary Material), no outliers were removed from the analysis and the entire sample’s result is shown in the following.

**Table 1:** Group comparison between resilient and non-resilient participants, calculated using Mann–Whitney U test^a^ (effect size=Cohen’s d) or Chi-square test^b^ (effect size = Phi): sex, age, handedness, SECPT-time, alcohol, coffee, nicotine, oral contraception, cycle phase

Variable	*P* value	Effect size	Resilient (*N* = 24)	Non-resilient (*N* = 24)
SECPT-time (min: sec)	0.104^a^	0.36	Mean (*SD)* = 2:59 (0:01)	Mean (*SD)* = 2:54 (0:09)
Sex	0.365^b^	0.13	*N* = 14 female; *N* = 10 male	*N* = 17 female; *N* = 7 male
Age (years)	0.439^a^	0.23	Mean (*SD)* = 34.5 (9.32)	Mean (*SD)* = 31.6 (5.52)
Handedness	1.000^b^	0.00	*N* = 22 right; *N* = 2 left	*N* = 22 right; *N* = 2 left
Alcohol	0.763^b^	0.04	*N* = 8 user; *N* = 16 non-user	*N* = 9 user; *N* = 15 non-user
Coffee	0.156^b^	0.20	*N* = 20 < 400ml; *N* = 4 > 400 ml	*N* = 23 < 400ml; *N* = 1 > 400 ml
Nicotine	0.220^b^	0.17	*N* = 22 non-user; *N* = 2 user	*N* = 19 non-user; *N* = 5 user
Oral contraception	0.613^b^	0.09	*N* = 11 non-user; *N* = 3 user	*N* = 12 non-user; *N* = 5 user
Cycle phase	0.930^b^	0.02	*N* = 5 follicular; *N* = 7 luteal; *N* = 1 menopausal; *N* = 1 unregular	*N* = 6 follicular; *N* = 9 luteal; *N* = 2 unregular

**Table 2:** Descriptives: salivary cortisol, subjective stress and State Anger

	All participants (*N* = 48)		Resilient (*N* = 24)		Non-resilient (*N* = 24)	
	Mean (SD)	Range	Mean (SD)	Range	Mean (SD)	Range
*Salivary cortisol log10 (nmol/L)*						
T0	0.45 (0.25)	− 0.09 to 1.05	0.45 (0.26)	− 0.09 to 1.05	0.44 (0.24)	− 0.02 to 0.95
T1	0.81 (0.33)	0.19 to 1.44	0.87 (0.34)	0.28 to 1.44	0.72 (0.30)	0.19 to 1.22
T1-T0	0.36 (0.32)	− 0.32 to 0.98	0.43 (0.36)	− 0.32 to 0.98	0.28(0.28)	− 0.16 to 0.83
*Subjective stress (VAS)*						
T0	2.42 (2.02)	0.00 to 7.16	1.78 (1.64)	0.00 to 6.43	3.00 (2.18)	0.00 to 7.16
T1	2.60 (2.17)	0.00 to 8.00	2.11 (1.39)	0.00 to 5.30	3.05 (2.65)	0.00 to 8.00
T1-T0	0.18 (1.43)	− 4.61 to 4.21	0.33 (1.38)	− 4.61 to 2.32	0.05 (1.48)	− 2.42 to 4.21
*State anger (STAXI-II)*						
T0	16.07 (2.57)	15.00 to 30.00	15.45 (1.10)	15.00 to 19.00	16.63 (3.33)	15.00 to 30.00
T1	15.98 (1.92)	15.00 to 24.00	15.55 (1.01)	15.00 to 19.00	16.38 (2.43)	15.00 to 24.00
T1-T0	− 0.09 (2.86)	− 15.00 to 7.00	0.09 (1.23)	− 4.00 to 2.00	− 0.25 (3.81)	− 15.00 to 7.00

### Physiological response: salivary cortisol

**Fig. 2:**
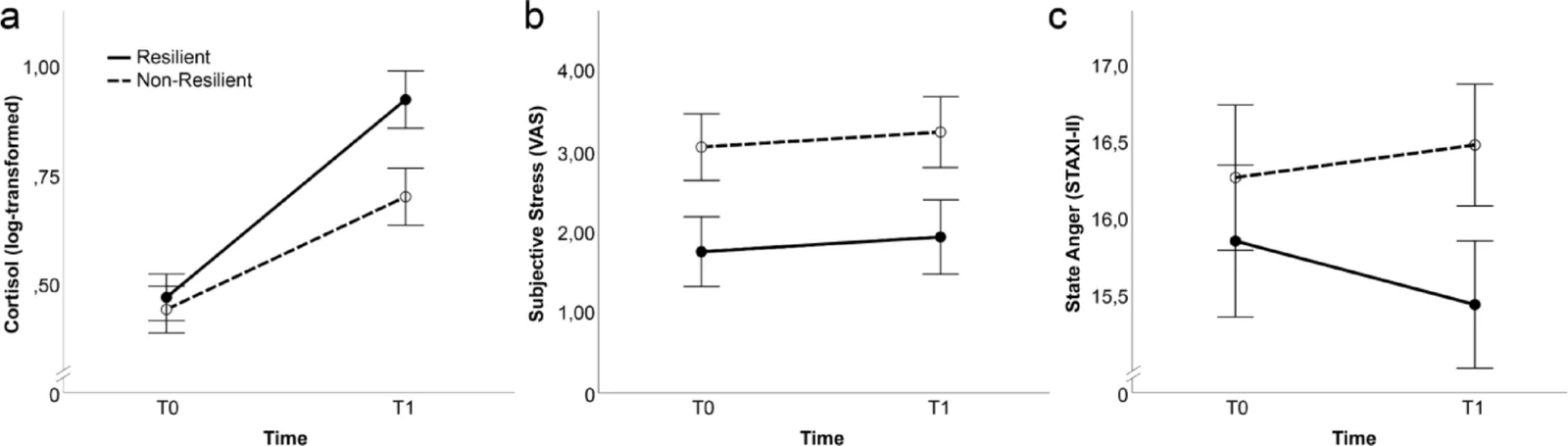
Acute stress response in resilient and non-resilient groups, analyzed using a repeated measures ANOVA with between-subject factor group and within-subject factor time, showing **a** salivary cortisol in nmol/L (log10-transformed), **b** subjective stress measured using a Visual Analogue Scale (VAS), and **c** state anger assessed with the State-Trait Anger Expression Inventory-2 (STAXI-II). Error bars represent ± 1 standard deviation. In **c**, the minimum value for the variable State Anger is 15. Note that cortisol levels did not differ between groups at baseline (t(46) = 0.47, *p*= 0.640; Cohen’s d = 0.08)

Neither the main effect of *time* (*F*(1,45) = 3.491, *p*=0.068, η_p_^2^ = 0.072) nor the main effect of *group* (*F*(1,45) = 2.955, *p*=0.092, η_p_^2^ = 0.062) was significant. However, there was a significant interaction of *time x group* (*F*(1,45) = 4.252, *p*=0.045, η_p_^2^ = 0.086), indicating that cortisol reactivity over time differed between groups, with the resilient group showing higher elevation of cortisol level after stress induction (Fig. 2a).

**Fig. 3:**
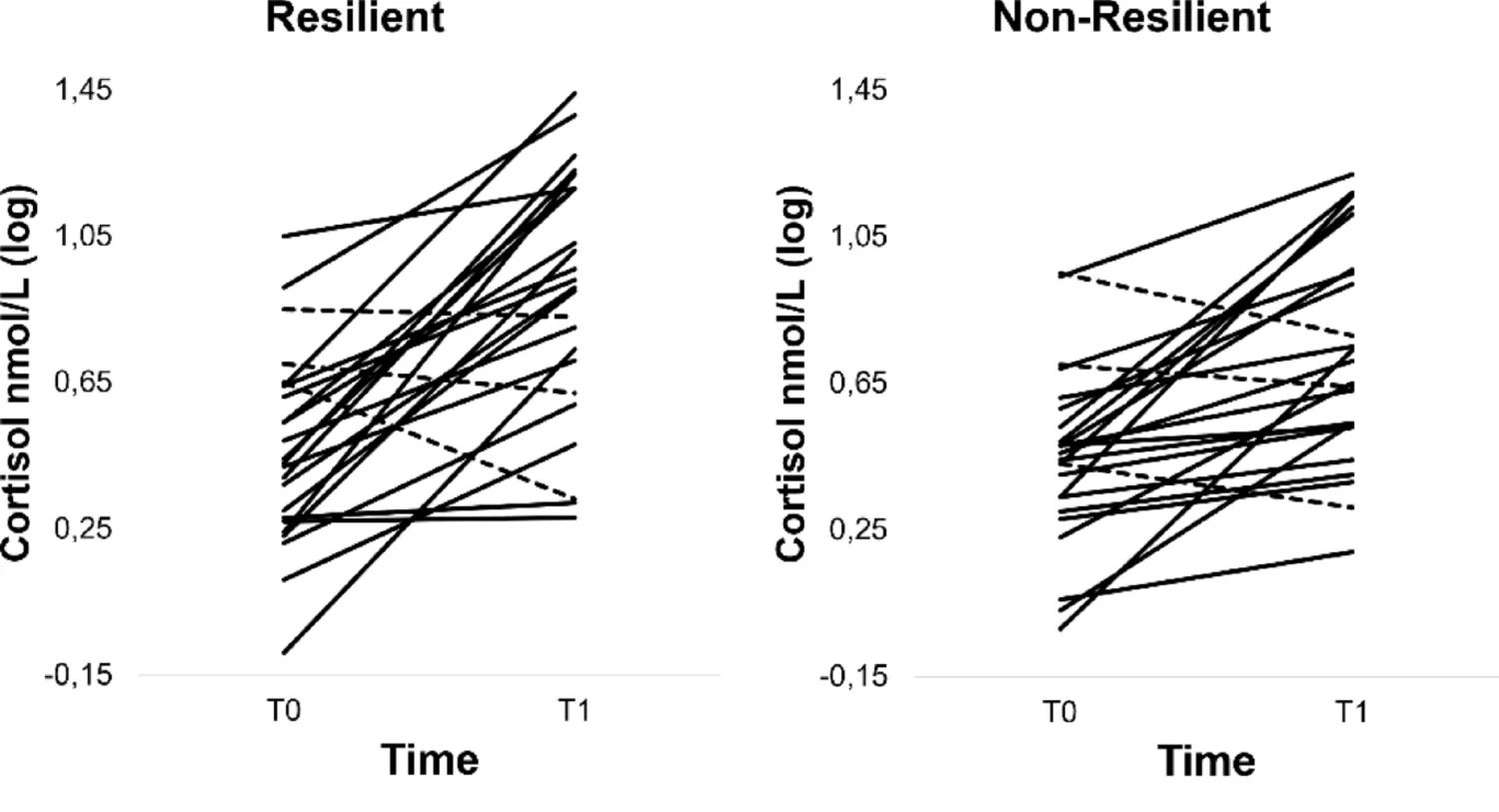
Individual salivary cortisol trajectories in nmol/L (log10-transformed) over time, separated by group, with non-responders (T1-T0 < 0) indicated by dotted lines

Spaghetti plots showing individual cortisol trajectories over time (Fig. 3) indicate lower cortisol reactivity in the non-resilient group. Non-responders (defined as T1-T0 < 0; indicated by dotted lines in Fig. 3) were equally distributed across both groups (3 resilient / 3 non-resilient).

### Subjective response: subjective stress, State Anger

Regarding subjective stress, repeated measures ANOVA showed significant main effects of *time* (*F*(1,43) = 4.640, *p*=0.037, η_p_^2^ = 0.097) and *group* (*F*(1,43) = 4.780, *p*= 0.034, η_p_^2^ = 0.100), but no significant interaction of *time x group* (*F*(1,43) = 0.000, *p*= 0.993, η_p_^2^ = 0.000), shown in Fig. 2b.

Concerning state anger, the main effect of *time* was significant (*F*(1,43) = 16.769, *p*< 0.001, η_p_^2^ = 0.281). Neither the main effect of *group* (*F*(1,43) = 1.963, *p*= 0.168, η_p_^2^ = 0.044) nor the interaction of *time x group* (*F*(1,43) = 0.658, *p*= 0.442, η_p_^2^ = 0.015) was significant (Fig. 2c).

### Correlations and exploratory findings

**Fig. 4:**
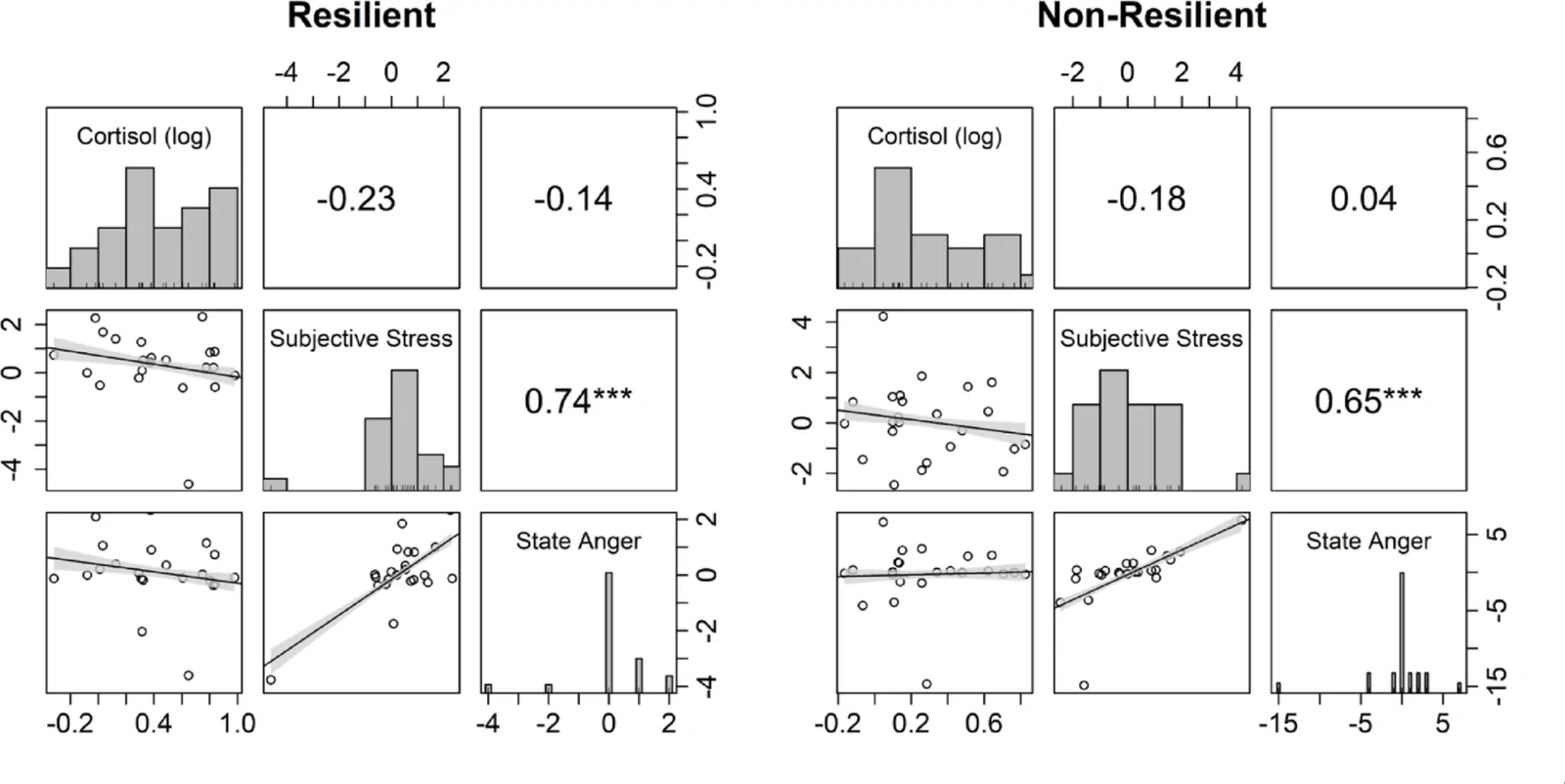
Correlations of difference scores (T1-T0) for salivary cortisol, subjective stress, and State Anger, separated bygroups. *p< 0.05; **p< 0.01; ***p< 0.001

Positive correlations were observed between difference scores (T0-T1) for subjective stress and State Anger, both in the resilient (*ρ* = 0.74, *p*< 0.001) and in the non-resilient group (*ρ* = 0.65, *p*< 0.001), presented in Fig. 4. The across-all correlation can be found in the Supplementary Material.

Exploratory correlation analyses revealed no significant associations between salivary cortisol and hair cortisol concentration (HCC), neither across all participants nor within groups (all *p-values* > 0.05). The lowest p-values observed were 0.353 (total sample), 0.198 (resilient group), and 0.249 (non-resilient group). Full details are provided in the Supplementary Material.

## Discussion

In the StReak study, we investigated the relationship between resilience and the acute stress response by testing whether resilient and non-resilient individuals differ in their physiological and subjective response to acute experimental stress. The SECPT was used as acute stressor, salivary cortisol as measure of the physiological response and subjective stress and State Anger as indicators of the subjective response. The StReak study was designed to complement the long-term assessment of resilience in the LORA study by capturing short-term stress responses, assuming corresponding differences between resilient and non-resilient individuals. Hypotheses were formulated non-directionally, as several possible outcomes were conceivable.

The present findings provide preliminary indications of differences between resilient and non-resilient individuals in terms of acute cortisol reactivity. Specifically, non-resilient individuals exhibited what we refer to as a “cortisol hypo-response,” operationalized as the comparatively lower change in cortisol levels between baseline (T0) and post-stress measurement (T1) relative to resilient individuals, as illustrated in Fig. 2a. No significant group differences were observed in baseline cortisol levels. These findings are consistent with our first hypothesis (1) regarding group differences in the acute physiological stress response.

In contrast to the observed group differences in cortisol reactivity, subjective stress ratings did not differ in terms of reactivity between groups. Therefore, our second hypothesis (2) was not confirmed. Nevertheless, non-resilient individuals exhibited higher overall subjective stress and State Anger scores than resilient individuals.

Given that cortisol was assessed at only two time points and the relatively small sample size, all findings should be interpreted with caution. Accordingly, the results are best understood as exploratory and suggestive rather than conclusive, particularly with respect to temporal dynamics. Overall, the results tentatively point toward a potential association between resilience and cortisol reactivity.

### Physiological response: salivary cortisol

#### Cortisol reactivity: mechanisms and literature integration

The observed group differences in cortisol reactivity raise questions about the underlying mechanisms driving these effects. On the one hand, lower resilience could be interpreted as a chronic state of increased stress sensitivity, potentially leading to long-term alterations of the HPA axis and a blunted cortisol response to acute stress. Such a downregulation may represent a compensatory mechanism to protect the organism from prolonged exposure to elevated cortisol levels. This interpretation is consistent with the observed cortisol hypo-response (i.e., a comparatively lower T0–T1 change in cortisol, see Limitations for discussion of alternative temporal patterns) in the non-resilient group in the present study. On the other hand, it is equally conceivable that pre-existing alterations in HPA-axis functioning may contribute to reduced stress adaptability and thereby lower resilience, due to an impaired physiological response to acute stressors. Since an increase in cortisol is considered a normative response to acute stress (He et al. 2021), the absence of such an adaptive response may indicate reduced flexibility of the stress system. This provides a potential explanatory framework for the observed pattern of cortisol reactivity in the non-resilient individuals in our sample. Thus, it remains unresolved whether non-resilient individuals exhibit cortisol hypo-reactivity as a consequence of lower resilience, or whether altered HPA-axis functioning contributes causally to reduced resilience. A reciprocal relationship between resilience and HPA-axis regulation is plausible.

In contrast to our findings, Mikolajczak et al. (2008) reported no significant group differences in cortisol reactivity between highly and less resilient individuals. However, highly resilient individuals showed lower baseline cortisol levels, whereas no baseline differences were observed in the present study.

Differences in subjective stress responses were also reported in that study, whereas no differences in subjective stress reactivity were found in our sample. These discrepancies may be explained by differences in resilience assessment, as Mikolajczak et al. used questionnaire-based measures and included only male participants.

Overall, prior evidence appears consistent with our findings regarding differences in cortisol reactivity, suggesting that resilient individuals may show higher cortisol reactivity to acute stress than non-resilient individuals.

In line with this, research on police officers shows that low cortisol reactivity is associated with increased vulnerability to chronic stress trajectories, whereas higher cortisol reactivity is linked to resilient trajectories (Galatzer-Levy et al. 2014). Moreover, PTSD-related studies have repeatedly reported cortisol hypo-reactivity in response to acute stress (Metz et al. 2020; Wichmann et al. 2017; Zaba et al. 2015), especially if the trauma occurred during childhood (Santa Ana et al. 2006). Lower cortisol levels or reactivity in the acute trauma phase may be predictive for the development (Delahanty et al. 2000) or intensity (Ehring et al. 2008) of PTSD symptoms, while reduced pre-trauma cortisol reactivity may predict increased vulnerability (Steudte-Schmiedgen et al. 2015).

These findings raise the question of whether heightened cortisol reactivity may serve not only as a predictive marker but also as a protective factor in stress-related adaptation. Thus, the present findings fit within a broader literature linking higher acute cortisol reactivity to adaptive stress responding.

Animal studies may provide additional insight, although definitions of resilience vary across species and paradigms (Colditz and Hine 2016; Russo et al. 2012). In animal husbandry, resilience may be more accurately reflected by recovery to the pre-stress state rather than by the dynamics of physiological parameters during stress exposure (Colditz and Hine 2016). Differences in salivary cortisol reactivity were observed between pigs with varying living conditions, with those in enriched environments showing lower reactivity than those in poorer conditions (Luo et al. 2022). These animal findings are consistent with the interpretation of reduced cortisol reactivity in the non-resilient group observed in the present study. Early-life maternal care has been shown to influence HPA-axis development and stress reactivity in adult rats across generations (Francis et al. 1999; Liu et al. 1997), with reduced corticosterone responses linked to increased maternal care. These findings highlight the importance of early environmental factors in shaping stress system development, although translation to human resilience requires caution.

#### Baseline cortisol and HPA-axis functioning

Notably, the present study found no group differences in baseline cortisol levels. This is noteworthy, given that many studies on PTSD patients report differences in the cortisol awakening response (CAR), which is a common indicator for HPA function (Chida and Steptoe 2009). The CAR was observed to be weaker in patients with PTSD than in healthy controls (Pan et al. 2018). In line with this, lower cortisol baseline levels were found in PTSD patients (Majewski et al. 2023). A high CAR was proposed as a potential protective factor for PTSD symptoms (Marin et al. 2019). This pattern mirrors the absence of baseline differences observed in the present study. Additionally, higher post-awakening cortisol has been linked to better cognitive outcomes, with resilience mediating this relationship (Rivas-Diaz et al. 2025). Overall, our findings suggest that resilience may be more strongly associated with acute cortisol reactivity than with baseline HPA-axis activity.

#### Recovery and habituation

There appears to be a link between high resilience and reduced cortisol release after repeated stress induction (Rösner et al. 2023) suggesting resilience as a predictor of habituation. Efficient recovery of cortisol levels reflects HPA feedback activity and is considered an indicator of healthy adaptive HPA-axis functioning (He et al. 2021). Taken together, this suggests potential group differences in HPA-axis flexibility, which may be reflected in the cortisol reactivity pattern observed in the present study.

An increased CAR was found to be linked to slower cortisol recovery after acute stress in healthy individuals (Degering et al. 2023). An enhanced HPA feedback function was found in PTSD patients (Morris et al. 2012), suggesting that resilient and non-resilient individuals might also differ in this aspect.

### Subjective response: subjective stress, State Anger

In contrast to the findings for cortisol reactivity, no group differences emerged in the reactivity of subjective stress and State Anger. Thus, our second hypothesis (2) was not supported. Nevertheless, non-resilient individuals exhibited higher overall levels of subjective stress and State Anger compared to resilient individuals.

This pattern is consistent with prior evidence linking elevated subjective stress to altered cortisol dynamics (Buzgoova et al. 2020). Moreover, differences in recent stress exposure, which were not assessed in the present study, may further shape acute stress responses (Dalile et al. 2022) and should be considered in future research.

Notably, despite previous reported group differences in long-term subjective stress perception (LORA study), no corresponding differences were observed in short-term subjective stress reactivity in the present experimental setting. This discrepancy may reflect the distinction between chronic stress exposure over years and acute responses to experimentally induced stress. In addition, the SECPT combines physical and social stressors, whereas everyday stress experiences are primarily psychological in nature, which may further account for the absence of group differences in acute subjective reactivity. Future studies using alternative stress paradigms (see limitations) may help to further clarify these discrepancies.

### Future directions

Future research should include additional measurement time points to assess recovery dynamics more precisely. Moreover, repeated exposure to the SECPT may allow investigation of habituation processes in relation to resilience. Since the two groups only differed regarding cortisol after stress induction, but not at baseline, future studies could focus on the relationship between resilience and CAR, which has been shown to be weaker in PTSD patients (Pan et al. 2018).

Further, individual differences such as reliability and study adherence may be relevant in the context of resilience. In the present sample, the non-resilient group showed more postponements of testing appointments, no-shows without cancellation, and dropouts. These behavioural differences appear to mirror the physiological differences in cortisol reactivity observed between groups. The concept of flexible self-regulation (Bonanno 2021) may provide a promising framework for understanding resilience, although its operationalization remains challenging (Bonanno et al. 2024). Assessing components of flexible self-regulation in our sample and relating them to cortisol reactivity could provide further insight into these mechanisms.

Follow-up assessments of the present cohort may provide further insight into the stability of these findings over time. Current GHQ scores were significantly higher in the non-resilient group, indicating more mental health problems in this group and supporting cluster stability.

### Sample composition and currentness of clusters

To address the potential concern regarding the temporal gap between cluster derivation (2017–2021) and the current assessment (2023), we examined whether the originally defined resilience-based groups remained valid at the time of the present study. Resilient and non-resilient participants were originally classified based on their longitudinal Stressor-Reactivity (SR) scores in the LORA study. Current group validity was assessed using General Health Questionnaire (GHQ) scores as well as measures of stressor exposure and perception (Life Events, Daily Hassles, and Perceived Stress Scale).

Overall, the results support the continued validity of the original cluster assignment. Specifically, non-resilient individuals showed significantly higher GHQ scores compared to resilient individuals, while no meaningful group differences were observed in current stressor exposure and perception. These findings suggest a relative stability of group membership over time despite the temporal gap between classification and follow-up assessment.

At the same time, it should be noted that four participants in the non-resilient group showed comparatively lower GHQ scores than the rest of the group. Analyses excluding these participants indicated that their removal attenuated the observed group difference in salivary cortisol, suggesting that these cases may have exerted an influence on the results. This highlights the potential impact of individual variability and outliers within relatively small subsamples.

Taken together, while the present findings generally support the current validity of the resilience-based group assignment, they also underscore the importance of considering temporal dynamics and individual deviations when interpreting longitudinally derived cluster classifications. Full details of these analyses are provided in the Supplementary Material.

### Limitations

While the present findings provide preliminary insights into the relationship between resilience and acute stress reactivity, several limitations should be considered when interpreting the results.

It should be noted that the operationalization of cortisol hypo-response is based on only two measurement points (T0 and T1). Accordingly, alternative temporal patterns cannot be ruled out. For example, non-resilient individuals might show a delayed increase in cortisol, whereas resilient individuals might show a decrease over time. This limits conclusions regarding the full temporal dynamics of HPA-axis reactivity in relation to resilience and indicates that the findings should be interpreted as exploratory. Future studies with higher temporal resolution are needed to capture these dynamics more precisely.

In addition, subjective stress ratings were assessed at only one post-stress time point. Although cortisol typically peaks approximately 20 min after stress induction (Gordis et al. 2006; Schwabe et al. 2008), this design may not fully capture the immediate subjective stress response. Additional post-stress assessments would therefore be beneficial in future work.

A further limitation is the exclusion of individuals with mental disorders, which may have introduced a selection bias by reducing representation of more vulnerable individuals, thereby limiting generalizability.

The relatively small sample size is a further limitation of this study, primarily due to the limited availability of eligible non-resilient participants within the source cohort. This restricts the generalizability of the findings and may reduce statistical power. The small number of eligible non-resilient participants reflects the nature of this cluster, which is the smallest group and characterized by higher indications of mental health problems, limiting inclusion in the present study.

Finally, the SECPT paradigm differs from other commonly used protocols such as the Trier Social Stress Test (TSST; Kirschbaum et al. 1993), particularly with regard to the social-evaluative component. However, as an extension of the Cold Pressor Test (CPT) (Hines and Brown 1936), the SECPT combines psychosocial and physical stress induction. In contrast to the SECPT (Minkley et al. 2014), the TSST may be affected by habituation effects upon repeated administration due to its reliance on a social-evaluative surprise component, although reliable cortisol responses have been reported when sufficient time intervals are applied between sessions (Petrowski et al. 2012). The SECPT was chosen due to its efficiency and its robust activation of the HPA axis (Schwabe et al. 2008). In addition, the SECPT was conducted using a slightly higher water temperature with 3–5 °C instead of the commonly used 0–2 °C range (Schwabe and Schächinger 2018) due to technical circumstances. Future studies may benefit from using the standard range for maximal comparability and reproducibility.

### Exploratory findings: salivary cortisol and hair cortisol

A paradoxical relationship between salivary and hair cortisol was found in patients with stress-related mental disorders: those who experienced childhood abuse had lower salivary but higher hair cortisol reactivity (Schalinski et al. 2015) compared to those without childhood abuse. This suggests that the stress system changes after experiencing severe stress, indicating the importance of distinguishing between acute and long-term physiological markers.

A weak negative association between resilience and hair cortisol has been reported (Xiang et al. 2023), which is not necessarily inconsistent with our findings given that salivary and hair cortisol reflect different physiological processes. Elevated hair cortisol has been associated with a mental health trajectory indicating acute dysfunction (Ahrens et al. 2022).

In the present study, exploratory analyses did not reveal significant associations between acute (salivary cortisol) and long-term (HCC) physiological stress measures. This may indicate that acute cortisol responses and cumulative cortisol secretion over time capture distinct aspects of stress physiology. However, conclusions are limited by the small subsample with available HCC data.

## Conclusion

In conclusion, the present study reveals a connection between resilience and the acute stress response: Non-resilient individuals showed significantly lower cortisol reactivity to acute experimental stress than resilient individuals. It remains to be clarified whether resilience is determinant for cortisol reactivity or if cortisol reactivity itself is a mechanism underlying resilience. Understanding resilience could contribute to the development of prevention strategies for stress-related mental health states that might promote the development of mental disorders. Identifying different patterns of cortisol reactivity may help to detect individuals at risk of developing stress-related disorders, as blunted cortisol stress reactivity appears to be associated with susceptibility to mental illness (Brenner et al. 2009; Burke et al. 2005). Consequently, knowledge of an individual’s cortisol reactivity to acute stress might be useful for supporting at-risk individuals. They may be offered psychoeducation, for example on personal resources, stress reduction tools, or even components of Bonanno’s proposed concept of flexible self-regulation (Bonanno 2021). This study provides valuable directions for future research that may help vulnerable individuals, since the observed link between resilience and cortisol stress reactivity is a promising step toward a better understanding of resilience.

## Supplementary Information

Below is the link to the electronic supplementary material.

Supplementary Material 1

## Data Availability

The data is not publicly available as this is an ongoing study. Materials and analysis code for this study are available by emailing the corresponding author.
