# Supplementary material for: Stress and resilience: cortisol hypo-response to acute stress in non-resilient individuals
Source: J Neural Transm (Vienna). 2026 Jul 3;133(9):2231–45. doi: 10.1007/s00702-026-03218-9 (PMC13687156; doi:10.1007/s00702-026-03218-9)
Supplement: Supplementary file 1 [file 702_2026_3218_MOESM1_ESM.docx]

**Supplementary Material**

for the manuscript entitled

**Stress and resilience: Cortisol hypo-response to acute stress in**

**non-resilient individuals**

submitted to Journal of Neural Transmission

Nadine D. Riske^1^, Charlotte Schenk^1^, Kira F. Ahrens^1,2^, Bianca Kollmann^7,4^, Danuta Weichert^3^, Nadine Steinbach^1^, Jana F. Burlage^1^, Florian Freudenberg^1^, Klaus Lieb^3,4^, Raffael Kalisch^4,6^, Oliver Tüscher^3,4,5,8,9^, Maren Schmidt-Kassow^1^, Andreas Reif^1^, Michael M. Plichta^1^

1 Goethe University Frankfurt, University Hospital, Department of Psychiatry, Psychosomatic Medicine and Psychotherapy, Germany

2 Goethe University Frankfurt, Cooperative Brain Imaging Center - CoBIC, Frankfurt, Germany

3 Department of Psychiatry and Psychotherapy, University Medical Center of the Johannes Gutenberg University, Germany

4 Leibniz Institute for Resilience Research, Mainz, Germany

5 Institute of Molecular Biology, Mainz, Germany

6 Neuroimaging Center, Focus Program Translational Neuroscience, Johannes Gutenberg University Medical Center Mainz, Germany

7 Central Institute of Mental Health, Department of Neuropsychology and Psychological Resilience Research, Mannheim, Germany

8 Department of Psychiatry, Psychotherapy and Psychosomatic Medicine University Medicine Halle (Saale) of the Martin Luther University Halle-Wittenberg (MLU), Germany

9 German Center for Mental Health (DZPG), partner site Halle-Jena-Magdeburg

**Corresponding Author:**

Nadine D. Riske, Department of Psychiatry, Psychosomatic Medicine and

Psychotherapy, University Hospital Frankfurt, Heinrich-Hoffmann-Straße 10, 60528 Frankfurt am Main, Germany, +49 15730741712, nadine.riske@gmx.de

**Supplementary Material**

1. **Participants**

Individuals who take systemic medication influencing the HPA axis were excluded from the study, except for five individuals taking L-thyroxine. The reason for this exception is that L-thyroxine is a hormone that is physiologically produced by the body. Its supplementation treats thyroid hormone deficiency, i.e. a hypothyroid metabolic state and thus rebuilds the physiological condition. However, it should be ensured that the observed effect is not driven by L-thyroxine, as all five individuals belonged to the non-resilient group.

Repeated measures ANOVA with SECPT-time and medication with L-thyroxine as covariates showed a significant main effect of *group* (*F*(1,44)=5.101, *p*=.029). The main effect of *time* was non-significant (*F*(1,44)=2.115, *p*=.153), such as the interaction of *time x group* (*F*(1,44)=3.774, *p*=.058). Additionally, we created spaghetti plots to illustrate the individual cortisol trajectories of these five individuals (Supplementary Fig. 1). None of them showed marginal cortisol trajectories, given that there were individuals reacting less and more than these five individuals. Given that L-thyroxine is a physiological hormone and the small sample size, these five individuals were included in the analyses.

Due to the limited availability of eligible participants, moderate coffee consumption (up to 600 ml/day) and moderate alcohol intake on the previous day were permitted. Moderate alcohol consumption was evenly distributed between groups (8 resilient, 9 non-resilient). Analyses indicated that inclusion of coffee and alcohol intake as covariates in the ANOVA models did not significantly affect the pattern of results. Baseline cortisol did not differ between participants with and without moderate alcohol consumption (t(46)=0.113, p=.911). In previous research examining salivary cortisol, two hours’ abstinence from alcohol was tolerated (Nathan et al., 2020).

**Supplementary Fig. 1** Individual salivary cortisol trajectories in µg/dl over time, for all participants and the non-resilient group, with individuals taking L-thyroxine indicated by orange lines and non-responders (T1-T0 < 0) indicated by dotted lines

2. **Acute vs. long-term Stress Response**

To compare the individuals’ acute stress response (assessed in the StReak study) with their long-term stress response (assessed in the LORA study), two more measures were added to calculations: prior assessed scalp hair cortisol concentration (HCC) data and current scores of the Perceived Stress Scale (PSS) (Cohen et al., 1983), both reflecting long-term stress response (Stalder & Kirschbaum, 2012). Thus, the acute and long-term stress response were evaluated both physiologically and mentally and tested for correlation using R (version 4.3.3), presented in Supplementary Fig. 2.

Hair samples were collected as part of the LORA study quarterly between 2017 and 2021, which is the same period cluster building was based on, a limitation discussed in 4.1. For more details about HCC processing and calculation, see Ahrens et al., 2022. Regarding the physiological response, salivary cortisol (acute response) and corresponding HCC levels (long-term response) of the same individuals were tested for correlation. HCC levels from 26 individuals (12 resilient/14 non-resilient) were available. Regarding the mental response, acute subjective stress scores, assessed via Visual Analogue Scale (VAS), were examined for association with same individuals’ PSS value which reflects long-term stress perception.

For the present HCC calculations, baseline HCC level (first sample) and mean of all available HCC values were used. Welch´s t-test revealed no significant group differences concerning HCC, neither for baseline nor for mean level. No significant correlations were found between hair cortisol and salivary cortisol, neither for all participants nor for both groups separately, except for predictable correlations within salivary cortisol and within HCC levels. The same pattern was observed for correlations between PSS and subjective stress scores.

**Supplementary Fig. 2** Correlations of physiological response (hair cortisol concentration = HCC and salivary cortisol = cortisol) and mental response (Perceived Stress Scale = PSS and subjective stress) separated by group and overall; *p<.05; **p<.01; ***p<.001

3. **Preliminary Data Screening: Identifying Outliers**

In total, 10 extreme (4 resilient/6 non-resilient) and 3 mild (2 resilient/3 non-resilient) partially overlapping outliers were identified. Mild outliers were tolerated. Thus, the following refers only to the extreme ones.

Concerning salivary cortisol, 2 values stood out at T0 (both non-resilient). No cortisol outliers occurred at T1 and T1-T0. For subjective stress, only one outlier (resilient) was found, regarding T1-T0. For State Anger, 9 outliers (4 resilient/5 non-resilient) were identified (T0: 4 resilient/2 non-resilient; T1: both non-resilient; T1-T0: 1 resilient/4 non-resilient; partially overlapping).

Additionally, following the open lab book prior established, baseline cortisol levels (T0) were compared to reference ranges for salivary cortisol differentiating between sex, age and hours after awakening (Miller et al., 2016). This comparison revealed *N*=7 values below the 5^th^ and above the 95^th^ percentile (4 resilient/3 non-resilient), including the two values identified through box plots. Salivary cortisol was analysed both with and without these outliers, and results remained stable with and without them. The same applies to subjective stress and State Anger analyses.

4. **Correlations: Salivary Cortisol, Subjective Stress, State Anger**

Regarding the full sample, positive correlations were found between difference scores (T0-T1) subjective stress and State Anger (*ρ*=.61, *p*<.001), presented in Supplementary Fig. 3.

**Supplementary Fig. 3** Correlations of difference scores (T1-T0) overall: salivary cortisol, subjective stress, State Anger; *p<.05; **p<.01; ***p<.001

5. **Currentness of Clusters (Verification of Group Assignment)**

The assignment of individuals to the resilient or the non-resilient group was conducted prior to the StReak study, since it is based on the LORA study period between 2017 and 2021. To validate whether this assignment is still accurate, current General Health Questionnaire (GHQ) scores were assessed. High GHQ scores (>23) indicate mental health problems. Additionally, measures for stressor exposure and perception (Daily Hassles (DH), Life Events (LE) and Perceived Stress Scale (PSS) value) were updated for the sample of the present study due to current interviews and analyzed using R (version 4.3.3).

The assignment to the resilient or non-resilient group appeared reasonable, since Welch´s t-test revealed significantly higher GHQ scores in the non-resilient group (*t*(20)=-2.119, *p*=.047), indicating greater mental health problems in this group. Regarding the non-resilient group, four individuals had lower GHQ scores than the rest of the group. Analyses of salivary cortisol were conducted both including (p=.02) and excluding (p=.063) these participants. The results indicate that the exclusion of these individuals reduces the strength of the observed group difference, although a difference is still suggested by the data. The groups did not differ significantly concerning LE, DH and PSS values, indicating that subjective and objective stress exposure was similar within the sample and group differences in reactivity to acute stress might be based on differences in resilience rather than on variations in stress exposure.

6. **Visual Analogue Scale (VAS)**

The Visual Analogue Scale (VAS) used in this study included the instruction “Please indicate how stressed you currently feel,” with the endpoints labeled as “not stressed” and “very stressed” (see Supplementary Fig. 4).

**Supplementary Fig. 4** Visual Analogue Scale (VAS) used for subjective stress assessment

**References**

Ahrens, K. F., Neumann, R. J., Werthern, N. M. von, Kranz, T. M., Kollmann, B., Mattes, B., Puhlmann, L. M. C., Weichert, D., Lutz, B., Basten, U., Fiebach, C. J., Wessa, M., Kalisch, R., Lieb, K., Chiocchetti, A. G., Tüscher, O., Reif, A., & Plichta, M. M. (2022). Association of polygenic risk scores and hair cortisol with mental health trajectories during COVID lockdown. *Translational Psychiatry*, *12*(1), 396. https://doi.org/10.1038/s41398-022-02165-9

Cohen, S., Kamarck, T., & Mermelstein, R. (1983). A global measure of perceived stress. *Journal of Health and Social Behavior*, *24*(4), 385–396.

Miller, R., Stalder, T., Jarczok, M., Almeida, D. M., Badrick, E., Bartels, M., Boomsma, D. I., Coe, C. L., Dekker, M. C. J., Donzella, B., Fischer, J. E., Gunnar, M. R., Kumari, M., Lederbogen, F., Power, C., Ryff, C. D., Subramanian, S. V., Tiemeier, H., Watamura, S. E., & Kirschbaum, C. (2016). The CIRCORT database: Reference ranges and seasonal changes in diurnal salivary cortisol derived from a meta-dataset comprised of 15 field studies. *Psychoneuroendocrinology*, *73*, 16–23. https://doi.org/10.1016/j.psyneuen.2016.07.201

Nathan, M. D., Wiley, A., Mahon, P. B., Camuso, J., Sullivan, K., McCormick, K., Srivastava, A., Albert, K., Newhouse, P., & Joffe, H. (2020). Hypothalamic-pituitary-adrenal axis, subjective, and thermal stress responses in midlife women with vasomotor symptoms. *Menopause (New York, N.Y.)*, *28*(4), 439–443. https://doi.org/10.1097/GME.0000000000001703.

Stalder, T., & Kirschbaum, C. (2012). Analysis of cortisol in hair--state of the art and future directions. *Brain, Behavior, and Immunity*, *26*(7), 1019–1029. https://doi.org/10.1016/j.bbi.2012.02.002
